# ARL6IP5 reduces cisplatin-resistance by suppressing DNA repair and promoting apoptosis pathways in ovarian carcinoma

**DOI:** 10.1038/s41419-022-04568-4

**Published:** 2022-03-15

**Authors:** Ji-Ye Kim, Entaz Bahar, Jung-Yun Lee, Sunhee Chang, Se Hoon Kim, Eun Young Park, Sung-Im Do, Hyonok Yoon, Hyun-Soo Kim

**Affiliations:** 1 Department of Pathology, Ilsan Paik Hospital, Inje University College of Medicine, Goyang, Korea; 2 Department of Pathology, Yonsei University College of Medicine, Seoul, Korea; 3 Department of Pathology, National Cancer Center, Goyang, Korea; 4 Department of Convergence Medical Science and Biochemistry, Institute of Health Sciences, Gyeongsang National University School of Medicine, Jinju, Korea; 5 College of Pharmacy, Research Institute of Pharmaceutical Sciences, Gyeongsang National University, Jinju, Korea; 6 Department of Obstetrics and Gynecology, Institute of Women’s Life Medical Science, Yonsei University College of Medicine, Seoul, Korea; 7 Biostatistics Collaboration Team, Research Core Center, National Cancer Center, Goyang, Korea; 8 Department of Pathology, Kangbuk Samsung Hospital, Sungkyunkwan University School of Medicine, Seoul, Korea; 9 Department of Pathology and Translational Genomics, Samsung Medical Center, Sungkyunkwan University School of Medicine, Seoul, Korea

**Keywords:** Prognostic markers, Ovarian cancer

## Abstract

Ovarian carcinoma (OC) is the most lethal gynecological malignancy due to frequent recurrence resulting from cisplatin-resistance. *ARL6IP5* is a novel gene implicated to suppress cisplatin-resistance by activating apoptosis and inhibiting DNA repair through XRCC1 and PARP1. We investigated the clinicopathological and prognostic significance of the immunohistochemical ARL6IP5 expression on 79 post-chemotherapy OC patient tissue samples; in vitro, the effect of *ARL6IP5* overexpression (OE) and knockdown (KD) on cancer hallmark functions and the effect of *ARL6IP5* on the expression of DNA repair and apoptosis-related proteins were observed in OC cells and their cisplatin-resistant (CisR) counterparts. ARL6IP5 expression was significantly associated with chemotherapeutic response and was an independent prognosticator of progression-free and overall survival of high-grade serous OC patients. *ARL6IP5*-OE decreased cellular proliferation, invasion, migration, adhesion, and increased apoptosis (*p* < 0.05); the opposite was observed for *ARL6IP5*-KD. Notably, *ARL6IP5*-OE reduced cisplatin-resistance of both OC and CisR OC cells, while *ARL6IP5*-KD increased cisplatin-resistance (*p* < 0.05). *ARL6IP5*-OE suppressed the expressions of DNA repair proteins and increased those of pro-apoptotic proteins; the opposite was observed for *ARL6IP5*-KD. The recombinant ARL6IP5 protein (rARL6IP5) had the greatest apoptotic effect among cisplatin and olaparib, in both OC and CisR OC cells; moreover, rARL6IP5 was the only single agent in CisR OC cells to retain higher apoptotic efficacy compared with control (*p* < 0.05), indicating that the apoptotic pathway influenced by rARL6IP5 remained effective in CisR OC cells compared to cisplatin and olaparib. In conclusion, we demonstrated that ARL6IP5 is an independent prognosticator of OC patients with cellular functions of a tumor-suppressor, possibly influencing the development of cisplatin-resistance and progression of OC cells through regulation of DNA repair and apoptosis. rARL6IP5 had significantly greater apoptotic efficacy compared to conventional chemotherapeutic agents in both OC and CisR OC cells, suggesting that ARL6IP5 may be a valuable novel chemotherapeutic against CisR OC.

## Introduction

Ovarian carcinoma (OC) is the most lethal gynecological malignancy, accounting for 4% of all cancers in women [1–3]. The current standard of treatment for patients with advanced-stage OC is cytoreductive surgery with platinum-based neoadjuvant or adjuvant chemotherapy [4]. However, despite advances in surgical and chemotherapeutic treatment, the 5-year survival rate of advanced OC patients is 35–40%, mainly due to the development of cisplatin-resistance [5]. Therefore, investigating the mechanism of chemoresistance and discovering novel therapeutic approaches on cisplatin-resistant (CisR) OC is necessary to improve patient survival.

Cisplatin is a widely used chemotherapeutic for treating many different types of malignancies including OC [4]. Cisplatin triggers apoptosis by inducing DNA damage through DNA cross-linking [5]. However, carcinoma cells develop multiple complex mechanisms to overcome cisplatin-induced apoptosis, thus conferring resistance to cisplatin [5, 6]. Two of the major systems activated in cisplatin-resistance are the DNA repair and anti-apoptosis signaling pathways.

X-ray repair cross-complementing gene 1 (XRCC1) is an essential component for several important DNA repair systems, including the base-excision repair (BER), nucleotide-excision repair, and microhomology-mediated end joining (MMEJ) repair of double strand breaks [7–9]. XRCC1 functions as a molecular scaffold protein, intimately involved in the coordination of DNA repair by interacting with other components, such as in auto-modification reactions of poly (ADP-ribose) polymerases 1 (PARP1) and DNA glycosylases. PARP1, whose inhibitor (olaparib) is now a part of the standardized therapy in selected OC patients, interacts with XRCC1 both functionally and physically and regulates XRCC1 [10]. There has been an accumulating body of work suggesting that XRCC1 influences cancer progression and cisplatin-resistance [11–13]. XRCC1-mediated MMEJ repair was found to result in the mutagenic chromosomal translocations leading to cancer. Moreover, XRCC1 plays an indispensable role in the repair of DNA adducts formed by cisplatin, supporting its importance in the development of cisplatin-resistance.

ADP ribosylation factor-like GTPase 6 interacting protein 5 (ARL6IP5) is a 21.6-kD, microtubule-associated protein encoded by the *ARL6IP5* gene. It contains a prenylated Rab acceptor motif, which regulates intracellular protein transport. Previous studies have reported that ARL6IP5 is involved in the regulation of intracellular protein transport and oxidative stress. Importantly, it has been shown to induce cell apoptosis, particularly through the endoplasmic reticulum (ER) stress response pathway [14]. Moreover, ARL6IP5 functions as a repair protein in oxidative stress-induced DNA single-strand breaks in fibroblasts through regulation of XRCC1 via the mitogen-activated protein kinase (MAPK) signal pathway and through the protection of XRCC1 from ubiquitination and degradation [15, 16]. ARL6IP5 was implicated as a potent inhibitor of cisplatin-resistance through the CK2-XRCC1 pathway in gastric carcinoma [17]. However, the biological role and effects of ARL6IP5 in OC has not been investigated.

In this work, we aimed to investigate the connection of ARL6IP5 in the development of cisplatin-resistance through clinicopathologic and survival analysis and in vitro studies of OC cells and their CisR counterparts. Our results demonstrated that ARL6IP5 is an independent prognosticator of ovarian high-grade serous carcinoma (HGSC) patients with cellular functions of a tumor-suppressor, possibly influencing the development of cisplatin-resistance and progression of OC cells through regulation of DNA repair and apoptosis. ARL6IP5 may be a valuable novel chemotherapeutic agent against CisR OC.

## Materials and methods

### Cell lines

The human normal ovarian cell line Hs823.Tc and OC cell lines, ES-2, OV90, TOV21G, CaOV-4, CaOV-3, TOV112D, SKOV3, and OVCAR-3, were purchased from Korea BioTech Co., Ltd. (Seoul, Korea), a domestic distributor of the American Type Culture Collection (ATCC; Manassas, VA, USA). Cells were grown in 5% CO_2_ and 95% saturated humidity at 37 °C and cultured in their respective ATCC recommended medium, supplemented with fetal bovine serum (FBS; Gibco) and 1% penicillin–streptomycin (Sigma-Aldrich, St. Louis, USA) for optimal growth. CisR OV90 (OV90-CisR) and SKOV3 (SKOV3-CisR) cells were established using our previously described stepwise dose incremental method [18].

### Overexpression of ARL6IP5

A pcDNA3.1 plasmid encoding the human *ARL6IP5* and empty vector was obtained from GenScript (oHu24499; Piscataway, NJ, USA). Cells (0.5 × 10^5^ cells/well) were plated on a 24-well plate, propagated to 80% confluence at the time of transfection, and then transfected with Lipofectamine 2000 transfection reagent (Invitrogen, Thermo Fisher Scientific, Carlsbad, CA, USA) according to the manufacturer’s instructions. Briefly, 1 μg of ARL6IP5/empty vector mixed with 50 μL serum-free media, and 3 µL Lipofectamine 2000 (Invitrogen) was separately diluted in 50 µL serum-free medium. The solutions were combined, vortexed, and incubated for 5 min to allow the formation of complexes. Then, 50 µL of the complex were added to each well, and the plates were gently rocked by hand to disperse the treatment solution. Cells were then cultured for 48 h, and western blot analysis was used to verify *ARL6IP5* expression in the cells.

### siRNA transfection

siRNA transfections were performed according to the manufacturer’s instructions using Lipofectamine 2000 RNAiMAX transfection reagent (Invitrogen). Briefly, cells were seeded at a density of 0.5 × 10^5^ cells/mL in 24-well plate. The following day, cells were transfected with 10 pmol ARL6IP5 siRNA/non-targeting siRNA (Dharmacon, Lafayette, CO, USA) mixed with serum-free medium and incubated at 37 °C for 48 h, to ensure effective gene knockdown. The knockdown levels were monitored by western blotting at 48 h of post-transfection.

### Proliferation assay

EZ-cytox cell viability kits (DoGenBio Co., Ltd., Seoul, Korea) was used according to the manufacturer’s instructions. Briefly, cells (1 × 10^4^ cells/well) were plated in 96-well plates and incubated for 24, 48, or 72 h. After incubation, EZ-cytox was added to each well and incubated for 2 h. Then, the absorbance was recorded on a microplate reader (Synergy H1; BioTek, Winooski, VT, USA) at 490 nm.

### Source of rARL6IP5

rARL6IP5 (#ARL6IP5–9863H; Creative Biomart, NY, USA) is a full-length human ARL6IP5 protein, with a length of 1‒188 amino acids and was fused with a glutathione S-transferase-tag at N-terminus. According to the manufacturer’s information sheet, the protein was produced from *E.coli* transfection and purified by glutathione-sepharose. rARL6IP5 was added to the culture media for experiments. The rARL6IP5 used in this study was a glutathione S-transferase-tagged protein. Proteins with glutathione S-transferase (GST)-tag has been implicated to be responsible for cell translocation of large molecules, through an energy-dependent process involving endocytosis [19–21]. GSTs are also associated with transmembrane properties as they are known to be either membrane-bound microsomal or cytosolic [22]. It is likely that the rARL6IP5 in this study was delivered into the cell by the properties of GST.

### Determination of 50% inhibitory concentration

To determine 50% inhibitory concentration (IC_50_) values of cisplatin (*cis*-dichlorodiammine platinum (II); Sigma-Aldrich), olaparib (AZD2281; Adooq Bioscience, Irvine, CA, USA), and rARL6IP5, we measured the cell proliferation rate using EZ-cytox cell viability kits (DoGenBio Co., Ltd.). IC_50_ values were analyzed using GraphPad Prism Version 5.0 (San Diego, CA, USA).

### Invasion and migration assays

Briefly, the cells (2 × 10^4^ cells/well) in serum-free medium were added to the upper chamber of Transwell chambers (8-μm pore; Corning Inc., Corning, NY, USA) coated with Matrigel (Corning Inc.), and a medium supplemented with FBS was added to the lower chamber. After incubation for 24 h, the cells on the upper surface were removed using a cotton swab, whereas the cells invaded or migrated the lower chamber were fixed with 4% paraformaldehyde, stained with 0.1% crystal violet, dried, and photographed. The numbers of invading or migrating cells were measured using a microplate reader (Synergy H1; BioTek) at 570 nm.

### Wound-healing assay

Cells (1 × 10^5^ cells/well) were seeded into a 24-well plate and grown to monolayer of up to 70–80% confluence. The monolayer was scratched gently using a new 200-µL sterile pipette tip across the center of the well. After scratching, the well was washed with PBS, followed by replenishment with the fresh medium. Photos of the monolayer were taken on a microscope at 0 and 6 h. The gap distance was quantitatively evaluated using ImageJ (National Institutes of Health, Maryland, MD, USA).

### Adhesion assay

A CytoSelect cell adhesion kit utilizing a fibronectin-coated 48-well plate (Cell Biolabs, Inc., San Diego, CA, USA) was used. Briefly, 150 µL of cell suspension containing 1 × 10^6^ cells/mL in serum-free medium was added inside each well and incubated for 90 min in a cell culture incubator. Then medium was carefully discarded from the wells, and each well was gently washed with PBS, followed by the addition of cell stain solution and incubated for 10 min at room temperature. The stain solution was discarded, and washed with deionized water, air dried, and visualized under microscopy. The number of adherent cells were measured using a microplate reader (Synergy H1, BioTek) at 570 nm (Synergy H1, BioTek).

### Apoptosis assay

Hoechst 33342 staining was conducted to measure apoptotic cells treated with cisplatin. Briefly, the cells were seeded in 6-well plates with 2 × 10^5^ cells per well in the culture media and allowed to attach overnight. The cells were treated with cisplatin (50 µM and 100 µM) and incubated at 37 °C for 24 h. After incubation, the seeded cells were washed with PBS once, and then incubated with 5 μg/mL Hoechst 33342 for 15 min. Finally, the cells were washed twice with PBS and were observed under inverted fluorescence microscopy (Axioskop 2 plus microscope; Carl Zeiss, Oberkochen, Germany). The apoptotic nuclei were counted from five non-overlapping fields and expressed as a percentage of the total counted nuclei. Apoptosis was determined by terminal deoxynucleotidyl transferase dUTP nick end labeling (TUNEL) staining method according to the manufacturer’s protocol (#22844, AAT Bioquest, Inc. CA, USA). Briefly, the cells were grown in a 96-well plate treated with cisplatin (OV90 16.75 μM, SKOV3 20 μM), olaparib (OV90 32.68 μM, SKOV3 25 μM), ARL6IP5 (OV90 1 μg/mL, SKOV3 2.5 μg/mL), and their combination, incubated for 72 h at 37 °C and 5% CO_2_. After treatment, the cells were washed with PBS and fixed with 4% paraformaldehyde for 30 min at room temperature. Samples were then incubated with 50 μL of TUNEL working solution (0.5 µL of 100X Tunnelyte™ Red was added into 50 µL of reaction buffer) for 1 h at 37° C in a dark chamber. After removing the TUNEL working solution, the cells were washed with PBS, and were added 100 µL of reaction buffer, followed by measurement of fluorescence intensity (ex/em: 550 nm/590 nm) on a fluorescence microplate reader (Varioskan LUX; Thermo Fisher Scientific). TUNEL assay results were visualized with a fluorescence microscope (Axioskop 2 plus microscope; Carl Zeiss, Oberkochen, Germany).

### Western blotting

Western blotting was performed as described previously [18], using primary antibodies listed in Supplementary Table 1.

### Patient selection

We found 266 ovarian carcinoma patients who received at least one cycle of neoadjuvant chemotherapy (NAC) and interval debulking surgery (IDS) from 2006 to 2017. The inclusion criteria were as follows: (1) histological diagnosis of HGSC; (2) the FIGO stage II–IV diseases; and (3) availability of post-NAC tumor tissue samples and clinical data. The post-NAC samples, which were taken from the omental tumor tissues obtained with IDS, were available for 166 HGSC patients. Eighty-five patients whose tumors showed complete responses to NAC (i.e., no residual tumor) were excluded because the tumor tissues for immunohistochemical staining were insufficient. Finally, 79 ovarian HGSC patients were included. Chemotherapy response score (CRS) was determined by assessments of the interval debulking surgery specimen post-chemotherapy: CRS 1 indicates minimal or zero tumor response; CRS 2, appreciable tumor response amid a viable tumor that was easily identifiable in the omentum; and CRS 3, complete or near-complete response with zero or very little residual tumor up to a maximum 2 mm in size [23–25]. Chemotherapeutic response was defined as at least 6 months from the completion of last platinum-based treatment until the date of relapse; cisplatin-resistance was defined as progression <6 months after completing platinum-based chemotherapy [26].

### Pathological evaluation and immunohistochemical staining

**Fig. 1:**
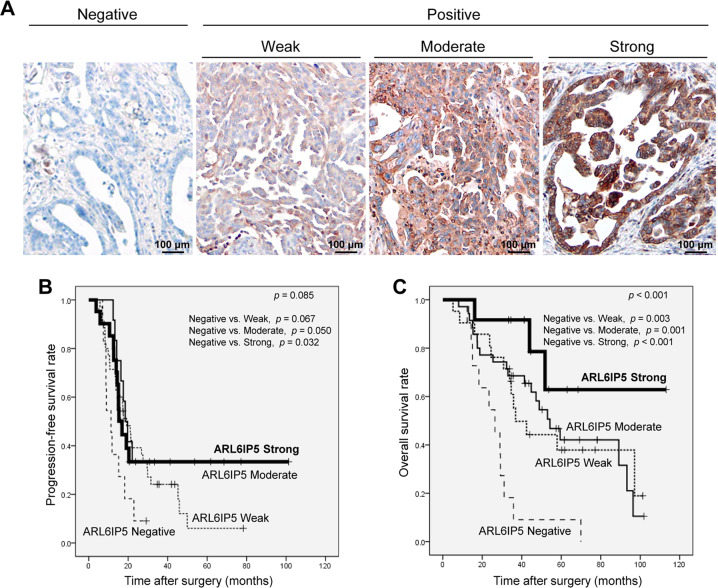
ARL6IP5 expression in ovarian HGSC patient tissue samples was significantly associated with increased survival. **A** Representative photomicrographs showing ARL6IP5 immunoreactivity (original magnification, ×20; scale bar, 100 µm). Kaplan–Meier plots for **B** PFS and **C** OS in 79 patients with ovarian HGSC.

All available hematoxylin and eosin-stained slides were examined, and a definite pathological diagnosis was made. The most representative slide for each case was selected for tissue microarray (TMA) construction and immunohistochemical staining. The most representative tumor region on the formalin-fixed paraffin-embedded (FFPE) tissue block was then marked, and a 3-mm tissue core sample was extracted using a punch machine and planted onto a recipient block. TMA blocks were sectioned 4-μm thick onto Super Frost Plus glass slides (Thermo Fisher Scientific). These sections were deparaffinized in xylene and rehydrated through graded alcohols. An automatic immunostaining instrument (Ventana Benchmark XT; Ventana Medical Systems) was used according to the manufacturer’s instructions. Antigen retrieval was performed using a cell conditioning solution (CC1; Ventana Medical Systems). The sections were incubated with polyclonal anti-ARL6IP5 antibody (1:100, Invitrogen). After the chromogenic visualization using an ultraView Universal DAB Detection Kit (Ventana Medical Systems), the slices were counterstained with hematoxylin, dehydrated in graded alcohols and xylene, and then embedded in mounting solution. Appropriate positive and negative controls were stained concurrently to validate the staining method. ARL6IP5 expression was analyzed using a semi-quantitative scoring method, as follows: 0, negative; 1, weak; 2, moderate; and 3, strong (Fig. 1A). Two board-certified pathologists (J.K. and S.C.) examined all ARL6IP5-immunostained slides blinded to clinical data. Discrepancies between the two pathologists were resolved by consensus.

### Three-dimensional (3D) spheroidal model and manual immunohistochemical staining

We used a combination of hanging drop and hydrogel scaffolds methods to generate in vitro three-dimensional (3D) spheroidal models [18, 27]. Briefly, 1.2% poly-2-hydroxylethyl methacrylate (Poly-HEMA) gel was prepared and coated on the bottom of 12-well plates and coated by 500 µL per well, air dried in a laminar flow cabinet. The cell aggregate sheets generated at 2 × 10^6^ cells/mL by hanging drops method were transferred to Poly-HEMA coated plate containing complete medium after 48 h. Then we treated the 3D spheroids with DMSO (control), cisplatin (OV90 15 µM; SKOV3 20 µM), olaparib (OV90 30 µM; SKOV3 25 µM) and rARL6IP5 (OV90 1 µg/mL; SKOV3 2.5 µg/mL) and incubated at 37 °C and 5% CO_2_ for 7 days with routine checking. After treatment period, the medium was aspirated off from each well and the spheroids were washed by adding PBS for 2 × 5 min. The 3D spheroids were fixed by adding 10% formalin. Following paraffin embedding, 3 μm thick sections were cut and mounted onto slides. All slides were kept at 58 °C for 60 min in a drying chamber, deparaffinized in xylene (#534056, Sigma-Aldrich) followed by rehydration in graded alcohols (#A405P-4, Thermo Fisher scientific). Subsequent slides were stained with hematoxylin and eosin (H&E) according to routine protocols or subjected to immunohistochemistry (IHC). For IHC, each slide was placed in IHC-Tek Epitope Retrieval Solution (#IW-1100, IHC World, Woodstock, MD, USA) and then placed in IHC-Tek Epitope Retrieval Steamer Set (#IW-1102, IHC World) for 20 min to utilize steaming method to achieve antigen unmasking on FFPE 3D spheroid sections. The slides were rinsed with IHC-Tek Washing Buffer (#IW-1201, IHC World) for 2 × 5 min. The slides were then blocked with IHC-Tek Peroxidase Blocking Solution (#IW-1300, IHC World) for 10 min and then rinsed with IHC-Tek Washing Buffer for 2 × 5 min. The slides were incubated in primary antibody at an appropriate dilution in IHC-Tek Antibody Diluent (#IW-1001, IHC World). IHC was performed using antibodies for ARL6IP5 (anti-rabbit, 1:300, MyBioSource, San Diego, CA, USA), XRCC1 (anti-mouse, 1:200, Thermo Fisher Scientific), PARP1 (anti-mouse, 1:200, Santa Cruz Biotechnology), Fas (anti-mouse, 1:200, Santa Cruz Biotechnology), cleaved caspase-3 (anti-mouse, 1:300, Santa Cruz Biotechnology). The slides were rinsed with IHC-Tek Washing Buffer for 2 × 5 min and subsequently incubated with secondary antibody (1:200) for 1 h. For the detection, IHC-Tek DAB Peroxidase substrate kit containing DAB chromogen concentrate (#IW-1600, IHC World) was used. The slides were then incubated in IHC-Tek Mayer’s Hematoxylin Solution (IW-1400, IHC World) for 10 min. The slides were dehydrated in 95 and 100% ethanol followed by xylene. Finally, the slides were air dried and were mounted using permanent mounting medium (#E01-18, Golden bridge International, Bothell, WA, USA).

### Statistical analysis

Student’s *t*-test and Fisher’s exact test were used for the continuous and categorical variables, respectively. One-way analysis of variance followed by Dunnett’s test was used for the analysis of at least three groups. For progression-free survival (PFS) and overall survival (OS), Kaplan–Meier survival curves and log-rank test were used. For the parameters with *p* < 0.1 in the univariable analysis, multivariable survival analysis was performed using the Cox proportional hazards model (95% CI) with the backward-stepwise elimination method. All statistical analyses were performed using IBM SPSS Statistics for Windows Version 25.0 (IBM Corporation, Armonk, NY, USA) and SAS Version 9.4 (SAS Institute Inc., Cary, NC, USA). Statistical significance was defined as *p* < 0.05.

## Results

### ARL6IP5 protein expression significantly correlated with chemotherapeutic response and increased survival in OC patients

Ovarian HGSC tissue from 79 patients obtained post-chemotherapy was analyzed to assess the relationship between ARL6IP5 expression and clinicopathologic parameters; the median follow-up duration was 32 (range: 2–113) months. 11.4% (9/79) of the HGSC cases were negative for ARL6IP5. ARL6IP5 expression was significantly different according to the level of chemotherapeutic response (*p* = 0.003; Table 1). ARL6IP5 expression correlated with a significant increase in both PFS and OS by log-rank and multivariable analysis (*p* < 0.05, Fig. 1 and Table 2). The median PFS and OS were 19 (range, 2–113) months and 44 (range, 3–113) months, respectively. PFS was not significantly correlated with ARL6IP5 staining by different intensities (*p* = 0.085). However, PFS was significantly different between the ARL6IP5 negative (average, 13.6 months; 95% CI, 9.6–17.6) and ARL6IP5 strong (average, 34.0 months; 95% CI, 20.1–47.9) groups, each respectively (*p* = 0.032, Fig. 1B). OS was significantly different among different ARL6IP5 staining intensities; average OS of the ARL6IP5-negative group was 27.7 months (95% CI, 18.3–37.1) while average OS of ARL6IP5 weak, moderate and strong group was 56.5, 59.4, and 86.4 months (95% CI, 40.7–72.2, 47.7–71.1, and 61.6–111.1, respectively), each respectively (*p* < 0.001; Fig. 1C). By multivariable analysis, positive ARL6IP5 (weak, moderate, and strong), CRS and FIGO stage, were independent prognosticators for PFS (hazard ratio, 0.45; 95% CI, 0.21–0.95, Table 2); positive ARL6IP5, CRS, and serum CA 125 level were independent prognosticators for OS (hazard ratio, 0.24; 95% CI, 0.11–0.52).

**Table 1:** Clinicopathological significance of ARL6IP5 expression in ovarian high-grade serous carcinoma patients.

Characteristic		ARL6IP5 expression				*p-*value
		Negative (%)	Weak (%)	Moderate (%)	Strong (%)	
Age (years)	≤60	7 (77.8)	10 (43.5)	22 (62.9)	8 (66.7)	0.249
	>60	2 (22.2)	13 (56.5)	13 (37.1)	4 (33.3)	
Serum CA 125 level (U/mL)	≤3600	5 (55.6)	17 (73.9)	25 (71.4)	6 (50.0)	0.405
	>3600	4 (44.4)	6 (26.1)	20 (28.6)	6 (50.0)	
Germline *BRCA* mutation status	Wild type	5 (55.6)	8 (34.8)	16 (45.7)	8 (66.7)	0.570
	VUS	1 (11.1)	3 (13.0)	4 (11.4)	2 (16.7)	
	Mutant	0 (0.0)	2 (8.7)	1 (2.9)	1 (8.3)	
	NA	3 (33.3)	10 (43.5)	14 (40.0)	1 (8.3)	
FIGO stage	II	3 (33.3)	11 (47.8)	12 (34.3)	4 (33.3)	0.719
	III-IV	6 (66.7)	12 (52.2)	23 (65.7)	8 (66.7)	
Residual tumor after IDS (cm)	≤0.5	6 (75.0)	19 (90.5)	26 (83.9)	10 (83.3)	0.763
	>0.5	2 (25.0)	9 (14.1)	5 (16.1)	2 (16.7)	
CRS	1	2 (22.2)	2 (8.7)	4 (11.4)	0 (0.0)	0.225
	2–3	7 (77.8)	21 (91.3)	31 (88.6)	12 (100.0)	
Chemotherapeutic response	Respondant	2 (22.2)	18 (78.3)	25 (71.4)	11 (91.7)	0.003^a^
	Resistant	7 (77.8)	3 (13.0)	8 (22.9)	0 (0.0)	
	Refractory	0 (0.0)	2 (8.7)	2 (5.7)	1 (8.3)	

*ARL6IP5* ADP ribosylation factor-like GTPase 6 interacting protein 5, *CA 125* cancer antigen 125, *CRS* chemotherapy response score, *FIGO* International Federation of Gynecology and Obstetrics, *IDS* interval debulking surgery, *NA* not applicable, VUS variant of unknown significance.

^a^Statistically significant.

**Table 2:** Prognostic significance of ARL6IP5 expression and clinicopathological characteristics in ovarian high-grade serous carcinoma patients.

Characteristic		Progression-free survival				Overall survival			
		Univariable		Multivariable		Univariable		Multivariable	
		HR (95% CI)	*p-*value	HR (95% CI)	*p*-value	HR (95% CI)	*p*-value	HR (95% CI)	*p*-value
Age (years)	≤60	1				1			
	>60	0.89 (0.52–1.50)	0.649			1.32 (0.75–2.34)	0.336		
Serum CA 125 level (U/mL)	≤3600	1				1		1	
	>3600	0.96 (0.56–1.64)	0.868			1.66 (0.92–2.99)	0.094	2.09 (1.14–3.84)	0.017^a^
Germline *BRCA* mutation status	Wild type	1	(0.842)			1	(0.196)		
	VUS	1.25 (0.44–3.62)	0.675			1.35 (0.39–4.66)	0.638		
	Mutant	0.87 (0.38–2.00)	0.738			0.27 (0.06–1.23)	0.091		
FIGO stage	II–III	1		1		1			
	IV	1.91 (1.10–3.30)	0.022^a^	1.93 (1.10–3.37)	0.022^a^	1.46 (0.80–2.67)	0.222		
Residual tumor after IDS (cm)	≤0.5	1				1			
	>0.5	1.78 (0.89–3.55)	0.104			0.80 (0.34–1.84)	0.592		
CRS	1	1		1		1		1	
	2–3	0.16 (0.07–0.38)	<0.001^a^	0.16 (0.07–0.40)	<0.001^a^	0.12 (0.05–0.27)	<0.001^a^	0.09 (0.04–0.22)	<0.001^a^
ARL6IP5 expression	Negative	1		1		1		1	
	Positive	0.35 (0.17–0.73)	0.005^a^	0.45 (0.21–0.95)	0.037^a^	0.24 (0.12–0.52)	<0.001^a^	0.24 (0.11–0.52)	<0.001^a^

*ARL6IP5* ADP ribosylation factor-like GTPase 6 interacting protein 5, *CA 125* cancer antigen 125, *CI* confidence interval, *CRS* chemotherapy response score, *FIGO* International Federation of Gynecology and Obstetrics, *HR* hazard ratio, *IDS* interval debulking surgery, *VUS* variant of unknown significance.

^a^Statistically significant.

### ARL6IP5 expression was lower in OC cells compared to normal ovarian cells

**Fig. 2:**
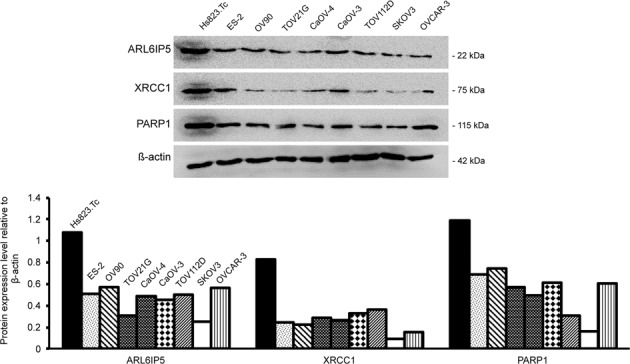
ARL6IP5, XRCC1, and PARP1 protein expression was low-throughout OC cell lines compared to normal ovarian epithelial cell. ARL6IP5, XRCC1, and PARP1 expression was analyzed by western blots in 8 OC cell lines, ES-2, OV-90, TOV21sG, CaOV-4, CaOV-3, TOV112D, SKOV3, OVCAR-3, and one normal ovarian cell line, Hs823.Tc.

ARL6IP5 expression was lower in all of 8 OC cell lines (ES-2, OV-90, TOV21G, CaOV-4, CaOV-3, TOV112D, SKOV3, and OVCAR-3; Fig. 2) compared to normal ovarian cell (Hs823.Tc). Expressions of DNA repair-associated proteins, XRCC1 and PARP1, were also lower in all OC cell lines compared to those of Hs823.Tc.

### ARL6IP5 suppressed tumorigenicity in OC cells

**Fig. 3:**
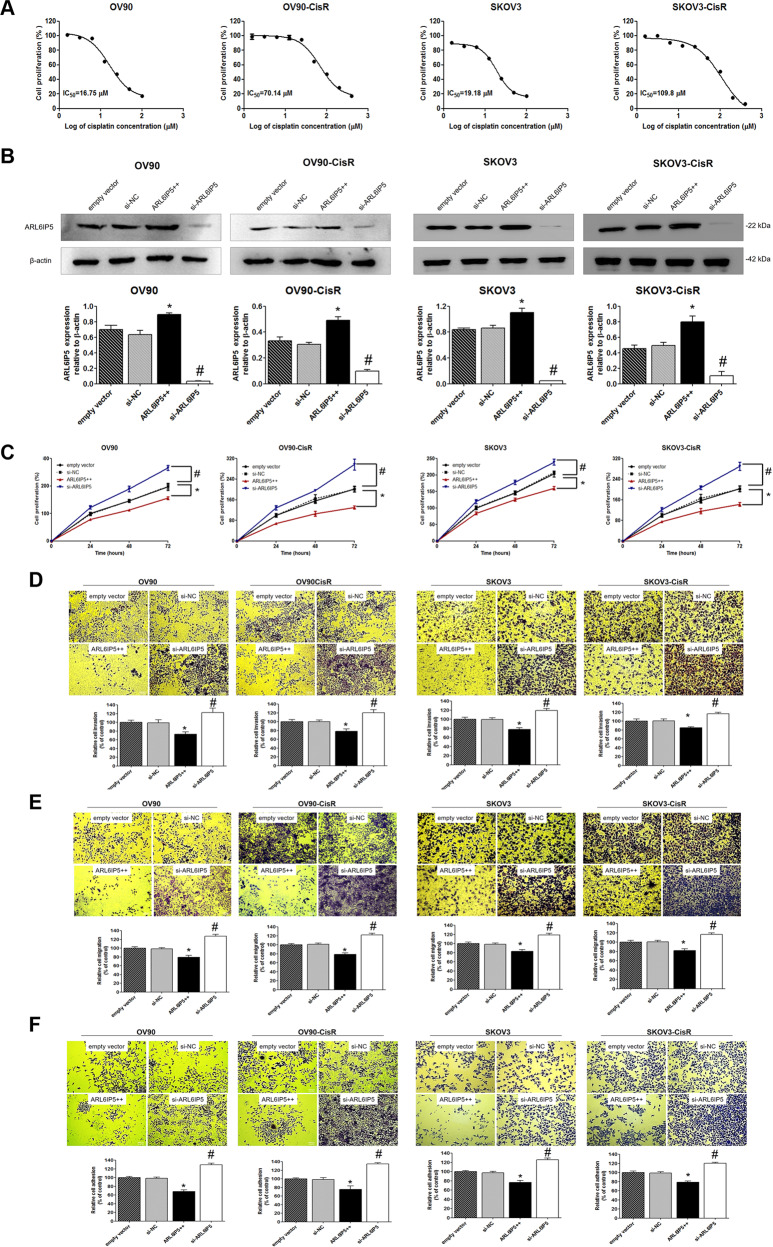
ARL6IP5 inhibited cellular proliferation, invasion, migration, and adhesion in OC and CisR OC cells. **A** CisR cells were generated from OC cells via gradual increments of cisplatin treatment for 8 months. Cell viability and IC_50_ of OC and CisR OC cells following exposure to cisplatin were determined. The IC_50_ of the resulting CisR cells were over five times greater than the parental OC cells. **B** OC and CisR OC cells were transfected with empty vector, si-NC (for negative control), pcARL6IP5 (ARL6IP5 + + ; for overexpression), and si-ARL6IP5 (for knockdown). Effects of *ARL6IP5* overexpression and knockdown on cellular **C** proliferation, **D** invasion, **E** migration, **F** adhesion in OC and CisR OC cells (original magnification, ×10; scale bar, 100 µm). *p < 0.05 compared with empty vector; ^#^*p* < 0.05 compared with si-NC.

IC_50_ values of cisplatin on OC cells and their CisR counterparts were evaluated from dose-response curves. The IC_50_ concentration of OV90-CisR and SKOV3-CisR was over 4–5 times greater than parental cells (70.14 µM vs. 16.75 µM and 109.8 µM vs. 19.18 µM, each respectively; Fig. 3A).

**Fig. 4:**
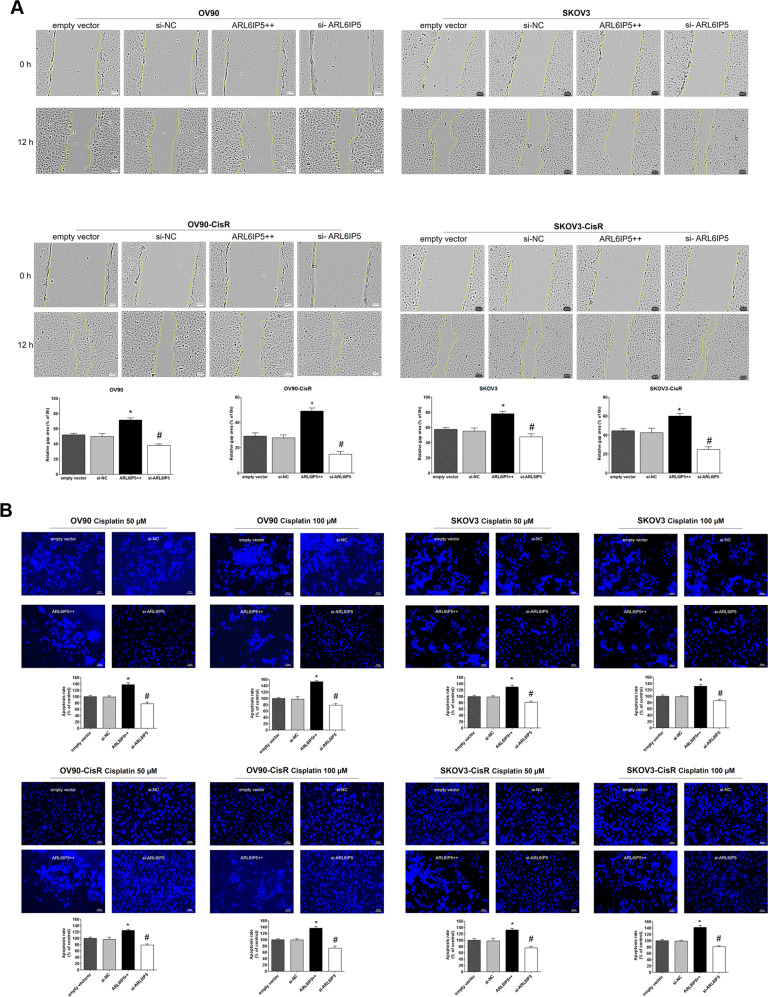
ARL6IP5 inhibited wound healing and apoptosis in OC and CisR OC cells. OC and CisR OC cells were transfected with empty vector, si-NC, pcARL6IP5 (ARL6IP5 + + ; for overexpression), and si-ARL6IP5 (for knockdown). Effects of *ARL6IP5* overexpression (ARL6IP5 + + ) and knockdown (si-ARL6IP5) on **A** wound healing and **B** apoptosis in OC and CisR OC cells. **A** Wound-healing assay was performed to detect the composite adhesion and migration abilities of OC and CisR OC cells. Time-lapse microscopy images of wound closure; dotted lines define the area lacking the cells (original magnification, ×10; scale bar, 100 µm). Quantification of the invaded wounded area after 12 h expressed as the percentage of gap area at 0 h. **B** Apoptosis assay of OC and CisR OC cells transfected with empty vector, si-NC, pcARL6IP5 (ARL6IP5 + + ) and si-ARL6IP5. Cells were treated with 50 or 100 µM of cisplatin, and the apoptotic rates were analyzed 24 h after cisplatin treatment (original magnification, ×20; scale bar, 50 µm). *p < 0.05 compared with empty vector; ^#^*p* < 0.05 com*p*ared with si-NC.

Effects of ARL6IP5 expression on cancer hallmark functions of OC cells and their CisR counterparts were examined using proliferation, invasion, migration, adhesion, wound healing, and apoptosis assays. Transfection of *ARL6IP5* plasmid markedly increased expression of the ARL6IP5 protein, whereas ARL6IP5 expression was significantly decreased after treatment with siRNA (*p* < 0.05, Fig. 3B). In both OC cells and their CisR counterparts, *ARL6IP5* overexpression (*ARL6IP5*-OE) resulted in significantly lower proliferation, invasion, migration, adhesion, and wound healing compared to empty vector at 24, 48, and 72 h (*p* < 0.05; Figs. 3C–F and 4A). In contrast, *ARL6IP5* downregulation (*ARL6IP5*-KD) resulted in significantly increased cancer hallmark functions than the scrambled negative control siRNA (si-NC) group at 24, 48, and 72 h in both OC cells and their CisR counterparts (*p* < 0.05, Figs. 3C–F and 4A). Apoptotic effect of ARL6IP5 was examined treating both OC cells and their CisR counterparts with 50 µM and 100 µM of cisplatin for 24 h. *ARL6IP5*-OE significantly decreased cell densities of viable non-apoptotic cells compared to empty vector, while *ARL6IP5*-KD significantly increased cell densities of viable cells in both OC cells and their CisR counterparts compared to si-NC (Fig. 4B). In other words, ARL6IP5 expression correlated with increased apoptotic effect.

### CisR cells had significantly lower basal ARL6IP5 and pro-apoptotic protein and significantly higher DNA repair protein expression compared to parental cells

**Fig. 5:**
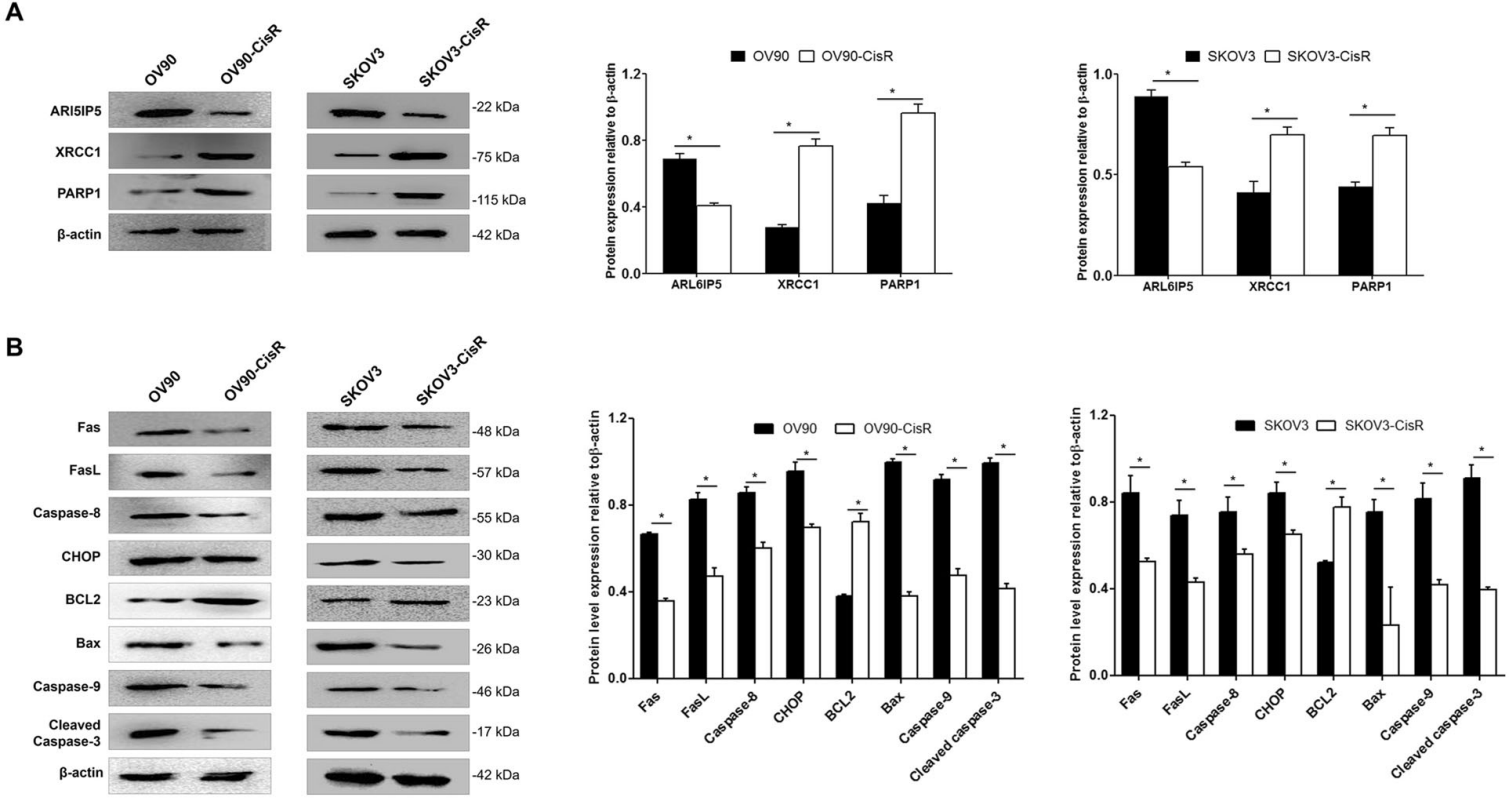
Basal ARL6IP5 expression was significantly decreased in OC cells compared to their CisR counterpart. Basal expression levels of **A** DNA repair-related proteins and **B** apoptosis-related proteins were analyzed by western blots. *p < 0.05.

CisR cells had significantly lower basal ARL6IP5 protein level compared to parental OC cells. Also, basal expression of DNA repair proteins (Fig. 5A) and apoptosis-related proteins (Fig. 5B) were significantly different between OC cells and their CisR counterparts. CisR cells had significantly higher basal DNA repair protein, i.e., XRCC1 and PARP1, expression compared to parental OC cells; basal pro-apoptotic proteins, i.e., Fas, FasL, Caspase-8, CHOP, Bax, Caspase-9, and Cleaved caspase-3, were significantly lower, whereas Bcl-2, the anti-apoptotic protein, was significantly higher in CisR cells than in parental OC cells (*p* < 0.05).

### ARL6IP5-OE increased cisplatin-response in OC cells by downregulating expression of DNA repair proteins and upregulating pro-apoptotic proteins

**Fig. 6:**
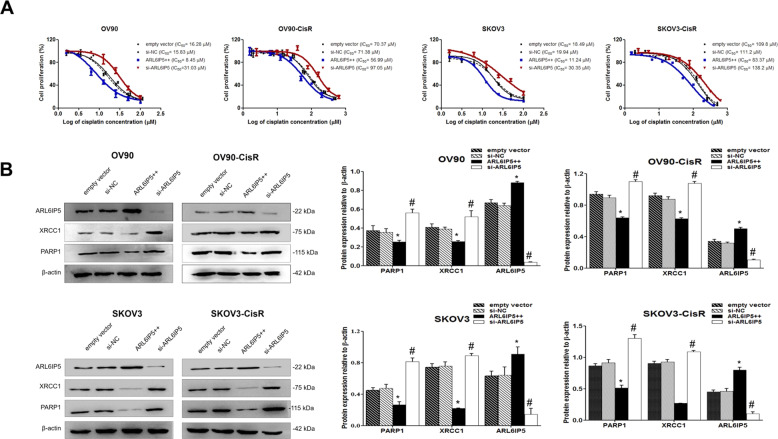
ARL6IP5 expression significantly reduced cisplatin-resistance and significantly decreased DNA repair protein expression. **A** Cell viability and IC_50_ of OC and CisR OC cells transfected with empty vector, si-NC, pcARL6IP5 (ARL6IP5 + + ; for overexpression), and si-ARL6IP5 (for knockdown) following exposure to cisplatin. **B** Expression of DNA repair-related proteins to determine the effect of *ARL6IP5* overexpression and knockdown were analyzed by western blots. *p < 0.05 compared with empty vector; ^#^*p* < 0.05 com*p*ared with si-NC.

**Fig. 7:**
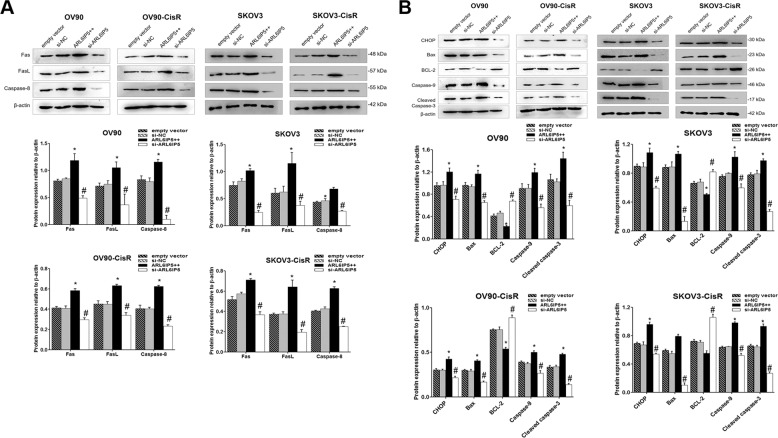
ARL6IP5 expression significantly reduced cisplatin-resistance and significantly increased pro-apoptotic protein expression. Expression of apoptosis-related proteins in the **A** extrinsic and **B** intrinsic pathways to determine the effect of *ARL6IP5* overexpression and knockdown were analyzed by western blots. *p < 0.05 compared with empty vector; ^#^*p* < 0.05 com*p*ared with si-NC.

To understand the effect of ARL6IP5 on cisplatin sensitivity of OC cells, the IC_50_ of empty vector, *ARL6IP5*-OE, si-NC, and *ARL6IP5*-KD in both OC cells and their CisR counterparts were calculated (Fig. 6A). IC_50_ of cisplatin in OC cells and their CisR counterparts were both significantly decreased in *ARL6IP5*-OE compared to empty vector (OV90, 8.45 µM vs. 16.28 µM; OV90-CisR, 56.99 µM vs. 70.37 µM; SKOV3, 11.24 µM vs. 18.49 µM; SKOV3-CisR, 83.37 µM vs. 109.8 µM; all *p* < 0.05) while IC_50_ of cisplatin was significantly increased in *ARL6IP5*-KD compared to si-NC (OV90, 31.03 µM vs. 15.83 µM; OV90-CisR, 97.05 µM vs. 71.38 µM; SKOV3, 30.35 µM vs. 19.94 µM; SKOV3-CisR 138.2 µM vs. 111.2 µM; *p* < 0.05). In other words, *ARL6IP5*-OE reduced cisplatin-resistance and *ARL6IP5*-KD increased cisplatin-resistance. *ARL6IP5*-OE significantly downregulated expression of DNA repair proteins, XRCC1 and PARP1 in both OC cells and their CisR counterparts compared to empty vector (*p* < 0.05). In contrast, *ARL6IP5*-KD had the opposite effect (Fig. 6B). *ARL6IP5*-OE in OC cells and their CisR counterparts significantly increased the expression of pro-apoptotic proteins in both extrinsic (Fas, FasL, and Caspase-8; Fig. 7A) and intrinsic (CHOP, Bax, and Caspase-9; Fig. 7B) pathways, including the main effector caspase, Cleaved caspase-3 compared to empty vector (*p* < 0.05). Furthermore, *ARL6IP5*-OE significantly decreased expression of the anti-apoptotic protein, Bcl-2 in both OC cells and their CisR counterparts compared to empty vector (*p* < 0.05); *ARL6IP5*-KD compared to si-NC, exhibited the opposite effect. These results indicate that ARL6IP5 induces apoptosis through the suppression of DNA repair and the activation of both intrinsic and extrinsic apoptotic pathways.

### rARL6IP5 treatment produced higher apoptotic rates than cisplatin and olaparib in both OC cells and their CisR lines

**Fig. 8:**
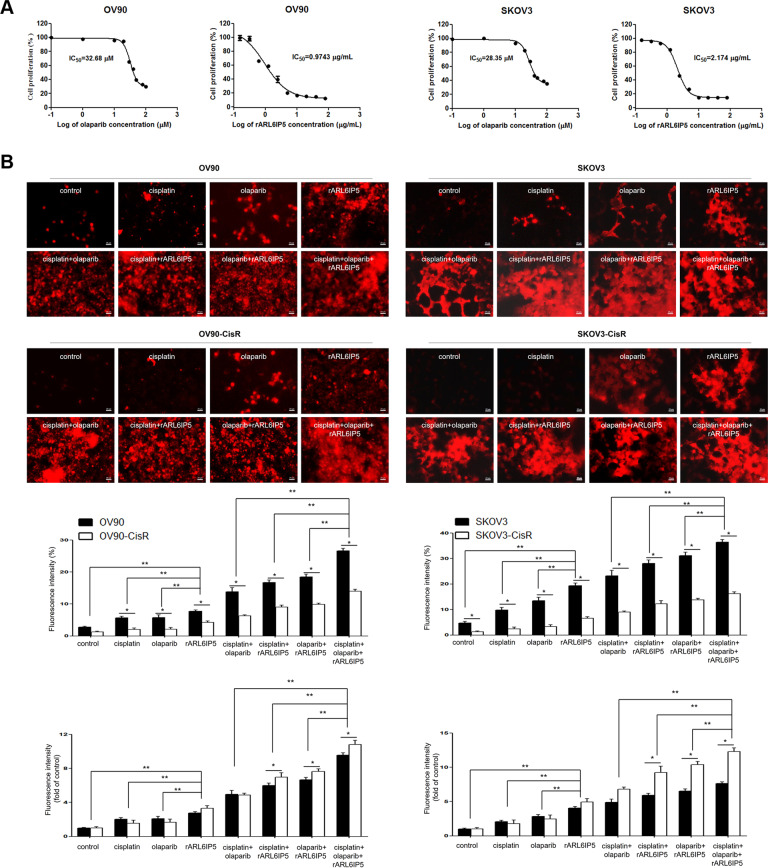
rARL6IP5 had significantly greater apoptotic efficacy compared to conventional chemotherapeutic agents (cisplatin and olaparib). **A** Cell viability and IC_50_ of OC and CisR OC cells following exposure to olaparib and rARL6IP5 were determined. IC_50_ of rARL6IP5 was <20% of that of olaparib in OC cells; in other words, rARL6IP5 was able to inhibit OC proliferation with <20% of the amount required by olaparib. **B** Therapeutic efficacy of cisplatin, olaparib, rARL6IP5, and their combinations in OC and CisR OC cells were evaluated by TUNEL assay (original magnification, ×40; scale bar, 20 µm). Apoptotic rate represented as fluorescence intensity percentage in OC and CisR OC cells. Apoptotic rate represented as fluorescence intensity as fold-change of control in OC and CisR OC cells. *p < 0.05, statistically significant; **p < 0.05, statistically significant in both OC and CisR OC cells.

The apoptotic effect of cisplatin, olaparib, rARL6IP5, and their combinations on OC cells was investigated using TUNEL assay, administering dosage of each respective IC_50_ values of each cell line (OV90, 15 μM, 30 μM, and 1.0 μg/mL; SKOV3, 20 μM, 25 μM, and 2.2 μg/mL; Figs. 3A and 8A). Cisplatin-resistance was confirmed by the significantly lower overall apoptotic rates in CisR cells than those of parental OC cells (*p* < 0.001; Fig. 8B and Supplementary Table 2). Cisplatin and olaparib had fluorescent intensities that were significantly different from control in parental OC cells. However, in the CisR OC cells the fluorescent intensities of cisplatin and olaparib were not significantly different from control; in other words, CisR OC cells had no apoptotic effect from the administration of cisplatin or olaparib (*p* < 0.05; Supplementary Table 3). Notably, rARL6IP5 had fluorescent intensities that were significantly higher than control, cisplatin, and olaparib in both OC cells and their CisR counterparts (*p* < 0.05), indicating that the apoptotic pathway influenced by rARL6IP5 remained relatively effective even in CisR cells compared to cisplatin and olaparib. rARL6IP5 had the highest fluorescent intensity among single agents with significant difference in both OC cells and their CisR counterparts (*p* < 0.05; Fig. 8B and Supplementary Table 2). Moreover, the combinations including rARL6IP5 (cisplatin + rARL6IP5, olaparib + rARL6IP5, and cisplatin + olaparib + rARL6IP5) had higher fluorescent intensity compared to combinations without rARL6IP5 (cisplatin + olaparib) by significant difference (*p* < 0.05). In terms of fold-change to control, rARL6IP5 was the only single agent with greater fluorescent intensity in CisR cells compared to parental OC cells (OV90, 3.30 vs. 2.78; SKOV3 5.0 vs. 4.08; Fig. 8B and Supplementary Table 2); in comparison, cisplatin and olaparib had lower fluorescent intensity in CisR cells compared to parental OC cells (OV90, 1.55 vs. 2.04 and 1.65 vs. 2.08; SKOV3, 1.8 vs. 2.1 and 2.5 vs. 2.8). Altogether, rARL6IP5 had greater apoptotic efficacy compared to the conventional chemotherapeutics of OC and demonstrated relatively preserved potency in the CisR OC cells compared to conventional chemotherapeutics. These findings indicate that ARL6IP5 is a powerful cell-death inducer that can reduce cisplatin-resistance in OC.

### rARL6IP5 treatment produced increased apoptotic marker expression and decreased DNA repair protein expression on 3D cultures of OC and CisR OC cells

The conventional monolayer cell culture usually does not replicate the physiologic conditions of the in vivo environment [28–30]. Therefore, we generated a 3D spheroid culture, recapitulating the organoid traits of the in vivo environment to compare the effect of rARL6IP5 treatment with those of cisplatin and olaparib. We applied H&E and immunohistochemical staining unto OC 3D cell cultures with different treatment conditions to investigate histological features together with markers of DNA repair and apoptosis (Supplementary Fig. 1). DNA repair activity was examined by PARP1 and XRCC1 protein expression. Apoptotic potential was examined by Cleaved caspase-3 and Fas protein expression. The H&E staining of 3D spheroids exhibited morphological features consistent with carcinoma cells; the 3D spheroids showed marked cellular atypia, including high nucleus to cytoplasm ratio, frequent mitosis, prominent nucleoli, and hyperchromatic nucleus. No significantly different morphological feature was observed between parental OC cells and CisR OC cells. DNA repair protein expressions were uniformly decreased in rARL6IP5 treated OC cells and their CisR counterparts compared to those treated with DMSO, cisplatin, or olaparib. Apoptotic protein expressions were also uniformly increased in rARL6IP5 treated OC cells and their CisR counterparts compared to those treated with DMSO (control), cisplatin, or olaparib. Altogether, based on these findings within the physiologically relevant organoid environment, rARL6IP5 has greater therapeutic potential compared to conventional chemotherapeutics.

## Discussion

In this work we demonstrated that ARL6IP5 is an important prognosticator and tumor-suppressor in OC, with potential to be a novel therapeutic. Clinically, ARL6IP5 expression correlated with chemotherapeutic response and influenced PFS and OS of HGSC. In vitro, we found that *ARL6IP5* expression was downregulated in all of 8 OC cell lines and further decreased in CisR cells compared to parental OC cells. Functional studies revealed that ARL6IP5 had tumor-suppressive effects; *ARL6IP5*-OE consistently decreased cellular proliferation, invasion, migration, adhesion, and wound healing. Moreover, *ARL6IP5*-OE reduced cisplatin-resistance and increased apoptosis in both OC cells and their CisR counterparts by suppressing DNA repair protein and increasing pro-apoptotic protein expression. Finally, even in CisR OC cells, rARL6IP5 had greater apoptotic efficacy compared to conventional chemotherapeutic agents, cisplatin and olaparib; therefore, rARL6IP5 may be a valuable chemotherapeutic, particularly advantageous in the setting of chemoresistance.

Previously, ARL6IP5 was reported to play a pivotal role in gastric carcinoma progression [17, 31–34]. Its tumor-suppressive functions associated with pro-apoptotic effect and DNA repair regulation have been reported in multiple malignancies, including hepatocellular carcinoma, leukemia, malignant melanoma, cervical carcinoma, and breast carcinoma [14, 35–38]. We found ARL6IP5 to be an independent prognosticator of both PFS and OS in ovarian HGSC patients. HGSC patients with ARL6IP5-negative expression had significantly shorter PFS and OS than those with ARL6IP5-positive expression. Our data suggest that ARL6IP5 is a tumor-suppressor that may be involved in the development, progression, and cisplatin-resistance of OC, in support of its clinical significance; therefore, ARL6IP5 expression may be used as a prognostic biomarker for OC.

We revealed several important connections of ARL6IP5 in cisplatin-resistance. ARL6IP5 expression was significantly associated with chemotherapeutic response, supported by in vitro findings; *ARL6IP5*-OE improved cisplatin-responsiveness in both OC cells and their CisR counterparts. Also, ARL6IP5’s differing basal expressions among normal cells, OC, and CisR OC suggests a connection to cisplatin-resistance. Basal ARL6IP5, XRCC1, and PARP1 expressions were significantly lower in OC cells compared to normal ovarian epithelial cells. Interestingly, ARL6IP5 expression was significantly lower in CisR cells compared to parental OC cells; whereas XRCC1 and PARP1 expression levels were significantly higher in CisR cells than in parental OC cells. DNA repair mechanism by XRCC1 and PARP1 to decrease cisplatin-responsiveness was activated after ARL6IP5 levels dropped from those of parental OC cells, confirmed by the cellular effects of *ARL6IP5*-KD. Previous studies on gastric carcinoma and fibroblasts, ARL6IP5 was found to be significantly lower in malignantly transformed cells compared to their normal counterparts [15, 17]. Notably, they discovered that ARL6IP5 had contrasting effects on XRCC1 in gastric carcinoma cells compared to their normal counterparts; on gastric carcinoma cells *ARL6IP5*-OE decreased XRCC1 levels, while in their normal counterparts *ARL6IP5*-OE increased XRCC1 levels. This could indicate that ARL6IP5 may drive the direction of DNA repair in a cell-specific manner. If contrasting effects can be confirmed between OC and its normal counterparts, the therapeutic value of ARL6IP5 increases because of its potential to selectively trigger cell-death on cancer cells while protecting normal cells; therefore, further studies are needed to confirm the full value of ARL6IP5 as a potential therapeutic agent.

Previous studies had indicated that ARL6IP5 serves as an important regulator of apoptosis [14, 39–42]. In esophageal carcinoma cells, *ARL6IP5*-OE induced apoptosis through Caspase-8 and Caspse-9, the key executioners of the extrinsic and intrinsic apoptosis pathways, respectively [39]. In breast carcinoma cells, *ARL6IP5* downregulation reduced apoptosis via the MAPK signaling pathway [42]. ARL6IP5 has also been reported to be localized in the ER, implicated in the ER stress-mediated apoptosis pathway of osteoblasts via the regulation of CHOP [14]. CHOP is an important mediator of apoptosis of the ER stress pathway, triggering apoptosis when the cell encounters prolonged ER stress [43]. We demonstrated that *ARL6IP5*-OE in both OC and CisR OC cells activated a wide spectrum of pro-apoptotic proteins of the extrinsic and intrinsic pathways, including mediators of ER stress, which supports the strong correlation between apoptotic rates and ARL6IP5 levels. Further studies are necessary to specify the dynamics of the molecules working in the extrinsic and intrinsic apoptotic pathways in relation to ARL6IP5 expression.

To the best of our knowledge, this is the first study to compare the apoptotic efficacy of rARL6IP5 with conventional chemotherapeutic agents; not only did rARL6IP5 have significantly greater apoptotic efficacy compared to cisplatin and olaparib, rARL6IP5 was the only single agent in CisR OC cells to retain significantly greater apoptotic efficacy compared to control. These findings imply that the apoptotic pathway influenced by rARL6IP5 remained relatively effective in CisR cells compared to conventional therapeutic agents. Altogether, these results suggest that ARL6IP5 has potential as a valuable therapeutic agent worthy of further study.

Intraperitoneal spread is another important cause of the low survival rate in OC patients [44]. Previous studies have shown that *ARL6IP5* downregulation caused increased cell migration and invasion in malignant melanoma, osteosarcoma, cervical carcinoma, esophageal carcinoma, and breast carcinoma [36, 39, 45]. We investigated the effects of *ARL6IP5*-OE and -KD in OC cells and their CisR counterparts. ARL6IP5 significantly inhibited tumorigenic abilities, such as proliferation, invasion, migration, adhesion, and wound healing of OC cells. Notably, the anti-tumorigenic ability of ARL6IP5 was also observed in CisR OC cells.

In conclusion, we report that ARL6IP5 is an independent prognosticator for both PFS and OS in ovarian HGSC patients influencing chemotherapeutic response. ARL6IP5 functioned as a tumor-suppressor, whose effects were confirmed in both OC and their CisR counterparts. ARL6IP5 had an apoptotic efficacy greater than those of conventional agents, through the activation of apoptotic pathways and suppression of DNA repair proteins. Unlike cisplatin and olaparib, whose apoptotic efficacy was not effective in CisR OC cells, rARL6IP5 had preserved apoptotic efficacy, effective even in CisR OC cells. We therefore suggest that ARL6IP5 may be a valuable therapeutic agent for CisR OC.

## Supplementary information

Supplementary Table 1

Supplementary Table 2

Supplementary Table 3

Supplementary Figure 1

Reproducibility checklist

Author Contribution Form

## Data Availability

The datasets generated and/or analyzed during this study are available from the corresponding author on reasonable request.
