# Supplementary material for: ARL6IP5 reduces cisplatin-resistance by suppressing DNA repair and promoting apoptosis pathways in ovarian carcinoma
Source: Cell Death Dis. 2022 Mar 15;13(3):239. doi: 10.1038/s41419-022-04568-4 (PMC8924236; doi:10.1038/s41419-022-04568-4)
Supplement: Supplementary file 1 — Supplementary Table 1 [file 41419_2022_4568_MOESM1_ESM.docx]

**Supplementary Table 1. List of primary antibodies**

| Antibody Name | Catalog Number | Supplier | Type of antibody |
| --- | --- | --- | --- |
| ARL6IP5 | MBS9212515 | MyBioSource | Rabbit polyclonal |
| XRCC1 | MA5-12071 | Thermo Fisher Scientific | Mouse monoclonal |
| PARP1 | sc-8007 | Santa Cruz Biotechnology | Mouse monoclonal |
| GRP78 | sc-13539 | Santa Cruz Biotechnology | Mouse monoclonal |
| CHOP | 2895 | Cell Signaling Technology | Mouse monoclonal |
| Bax | sc-20067 | Santa Cruz Biotechnology | Mouse monoclonal |
| Bcl-2 | sc-509 | Santa Cruz Biotechnology | Mouse monoclonal |
| FasL | SAB1402117 | Sigma Aldrich | Mouse monoclonal |
| Fas | sc-8009 | Santa Cruz Biotechnology | Mouse monoclonal |
| Caspase-8 | sc-56070 | Santa Cruz Biotechnology | Mouse monoclonal |
| Caspase-9 | sc-56073 | Santa Cruz Biotechnology | Mouse monoclonal |
| Cleaved caspase-3 | 9661 | Cell Signaling Technology | Rabbit polyclonal |
| β-actin | A5441 | Sigma-Aldrich | Mouse monoclonal |
