# Supplementary material for: ARL6IP5 reduces cisplatin-resistance by suppressing DNA repair and promoting apoptosis pathways in ovarian carcinoma
Source: Cell Death Dis. 2022 Mar 15;13(3):239. doi: 10.1038/s41419-022-04568-4 (PMC8924236; doi:10.1038/s41419-022-04568-4)
Supplement: Supplementary file 2 — Supplementary Table 2 [file 41419_2022_4568_MOESM2_ESM.docx]

**Supplementary Table 2. Quantification of apoptotic rates using TUNEL assay after treatment with cisplatin, olaparib, and rARL6IP5 and their combinations**

| Treatment | OV90 | | | OV90-CisR | | |
| --- | --- | --- | --- | --- | --- | --- |
|  | Average fluorescence intensity (%) ± SD | Average fold change to control ± SD | Relative ratio (%) to rARL6IP5 | Average fluorescence intensity (%) ± SD | Average fold change to control ± SD | Relative ratio (%) to rARL6IP5 |
| Control | 2.8 ± 0.2 | 1.0 ± 0.075 | 36.0 | 1.3 ± 0.2 | 1.0 ± 0.150 | 30.3 |
| Cisplatin | 5.7 ± 0.5 | 2.0 ± 0.173 | 73.4 | 2.0 ± 0.5 | 1.5 ± 0.368 | 46.9 |
| Olaparib | 5.8 ± 0.8 | 2.1 ± 0.286 | 75.0 | 2.1 ± 0.5 | 1.6 ± 0.368 | 50.0 |
| rARL6IP5 | 7.7 ± 0.4 | 2.8 ± 0.139 | 100.0 | 4.3 ± 0.4 | 3.3 ± 0.317 | 100.0 |
| Cisplatin + Olaparib | 13.8 ± 1.3 | 5.0 ± 0.464 | 178.7 | 6.3 ± 0.3 | 4.9 ± 0.216 | 147.8 |
| Cisplatin + rARL6IP5 | 16.8 ± 0.7 | 6.0 ± 0.266 | 216.5 | 9.0 ± 0.7 | 7.0 ± 0.507 | 211.4 |
| Olaparib + rARL6IP5 | 18.5 ± 0.8 | 6.7 ± 0.294 | 239.6 | 9.9 ± 0.4 | 7.6 ± 0.296 | 231.7 |
| Cisplatin + Olaparib + rARL6IP5 | 26.6 ± 0.8 | 9.6 ± 0.282 | 344.2 | 14.0 ± 0.6 | 10.8 ± 0.479 | 327.9 |
| Treatment | SKOV3 | | | SKOV3-CisR | | |
|  | Average fluorescence intensity (%) ± SD | Average fold change to control ± SD | Relative ratio (%) to rARL6IP5 | Average fluorescence intensity (%) ± SD | Average fold change to control ± SD | Relative ratio (%) to rARL6IP5 |
| Control | 4.8 ± 0.6 | 1.0 ± 0.119 | 24.5 | 1.3 ± 0.3 | 1.0 ± 0.204 | 20.1 |
| Cisplatin | 9.9 ± 1.0 | 2.1 ± 0.214 | 50.9 | 2.4 ± 0.7 | 1.8 ± 0.537 | 36.0 |
| Olaparib | 13.5 ± 1.3 | 2.8 ± 0.278 | 69.7 | 3.3 ± 0.7 | 2.5 ± 0.552 | 50.0 |
| rARL6IP5 | 19.4 ± 1.0 | 4.1 ± 0.205 | 100.0 | 6.6 ± 0.6 | 5.0 ± 0.477 | 100.0 |
| Cisplatin + Olaparib | 23.2 ± 2.2 | 4.9 ± 0.458 | 119.9 | 9.0 ± 0.4 | 6.8 ± 0.301 | 137.2 |
| Cisplatin + rARL6IP5 | 28.1 ± 1.2 | 5.9 ± 0.262 | 145.3 | 12.3 ± 1.2 | 9.3 ± 0.911 | 187.7 |
| Olaparib + rARL6IP5 | 31.1 ± 1.4 | 6.6 ± 0.290 | 160.8 | 13.8 ± 0.6 | 10.4 ± 0.460 | 209.6 |
| Cisplatin + Olaparib + rARL6IP5 | 36.4 ± 1.1 | 7.7 ± 0.226 | 188.0 | 16.3 ± 0.7 | 12.3 ± 0.544 | 247.7 |

Abbreviations: CisR, cisplatin-resistant; rARL6IP5, recombinant ARL6IP5; SD, standard deviation.
