# Supplementary material for: ARL6IP5 reduces cisplatin-resistance by suppressing DNA repair and promoting apoptosis pathways in ovarian carcinoma
Source: Cell Death Dis. 2022 Mar 15;13(3):239. doi: 10.1038/s41419-022-04568-4 (PMC8924236; doi:10.1038/s41419-022-04568-4)
Supplement: Supplementary file 3 — Supplementary Table 3 [file 41419_2022_4568_MOESM3_ESM.docx]

**Supplementary Table 3. Statistical significance of apoptotic rates between agents and their combinations.**

| Cell line | Agent | Control | Cisplatin | Olaparib | rARL6IP5 | Cisplatin + Olaparib | Cisplatin + rARL6IP5 | Olaparib + rARL6IP5 | Cisplatin + Olaparib + rARL6IP5 |
| --- | --- | --- | --- | --- | --- | --- | --- | --- | --- |
| OV90 | Control | ‒ | S | S | S | S | S | S | S |
|  | Cisplatin | S | ‒ | NS | NS | S | S | S | S |
|  | Olaparib | S | NS | ‒ | NS | S | S | S | S |
|  | rARL6IP5 | S | NS | NS | ‒ | S | S | S | S |
|  | Cisplatin + Olaparib | S | S | S | S | ‒ | S | S | S |
|  | Cisplatin + rARL6IP5 | S | S | S | S | S | ‒ | NS | S |
|  | Olaparib + rARL6IP5 | S | S | S | S | S | NS | ‒ | S |
|  | Cisplatin + Olaparib + rARL6IP5 | S | S | S | S | S | S | S | ‒ |
| OV90-CisR | Control | ‒ | NS | NS | S | S | S | S | S |
|  | Cisplatin | NS | ‒ | NS | S | S | S | S | S |
|  | Olaparib | NS | NS | ‒ | S | S | S | S | S |
|  | rARL6IP5 | S | S | S | ‒ | S | S | S | S |
|  | Cisplatin + Olaparib | S | S | S | S | ‒ | S | S | S |
|  | Cisplatin + rARL6IP5 | S | S | S | S | S | ‒ | NS | S |
|  | Olaparib + rARL6IP5 | S | S | S | S | S | NS | ‒ | S |
|  | Cisplatin + Olaparib + rARL6IP5 | S | S | S | S | S | S | S | ‒ |
| SKOV3 | Control | ‒ | S | S | S | S | S | S | S |
|  | Cisplatin | S | ‒ | NS | S | S | S | S | S |
|  | Olaparib | S | NS | ‒ | S | S | S | S | S |
|  | rARL6IP5 | S | S | S | ‒ | S | S | S | S |
|  | Cisplatin + Olaparib | S | S | S | S | ‒ | S | S | S |
|  | Cisplatin + rARL6IP5 | S | S | S | S | S | ‒ | NS | S |
|  | Olaparib + rARL6IP5 | S | S | S | S | S | NS | ‒ | S |
|  | Cisplatin + Olaparib + rARL6IP5 | S | S | S | S | S | S | S | ‒ |
| SKOV3-CisR | Control | ‒ | NS | NS | S | S | S | S | S |
|  | Cisplatin | NS | ‒ | NS | S | S | S | S | S |
|  | Olaparib | NS | NS | ‒ | S | S | S | S | S |
|  | rARL6IP5 | S | S | S | ‒ | S | S | S | S |
|  | Cisplatin + Olaparib | S | S | S | S | ‒ | S | S | S |
|  | Cisplatin + rARL6IP5 | S | S | S | S | S | ‒ | NS | S |
|  | Olaparib + rARL6IP5 | S | S | S | S | S | NS | ‒ | S |
|  | Cisplatin + Olaparib + rARL6IP5 | S | S | S | S | S | S | S | ‒ |

Abbreviations: CisR, cisplatin-resistant; NS, not significant; rARL6IP5, recombinant ARL6IP5; S, significant.
