# Supplementary figures and images for: ARL6IP5 reduces cisplatin-resistance by suppressing DNA repair and promoting apoptosis pathways in ovarian carcinoma
Source: Cell Death Dis. 2022 Mar 15;13(3):239. doi: 10.1038/s41419-022-04568-4 (PMC8924236; doi:10.1038/s41419-022-04568-4)

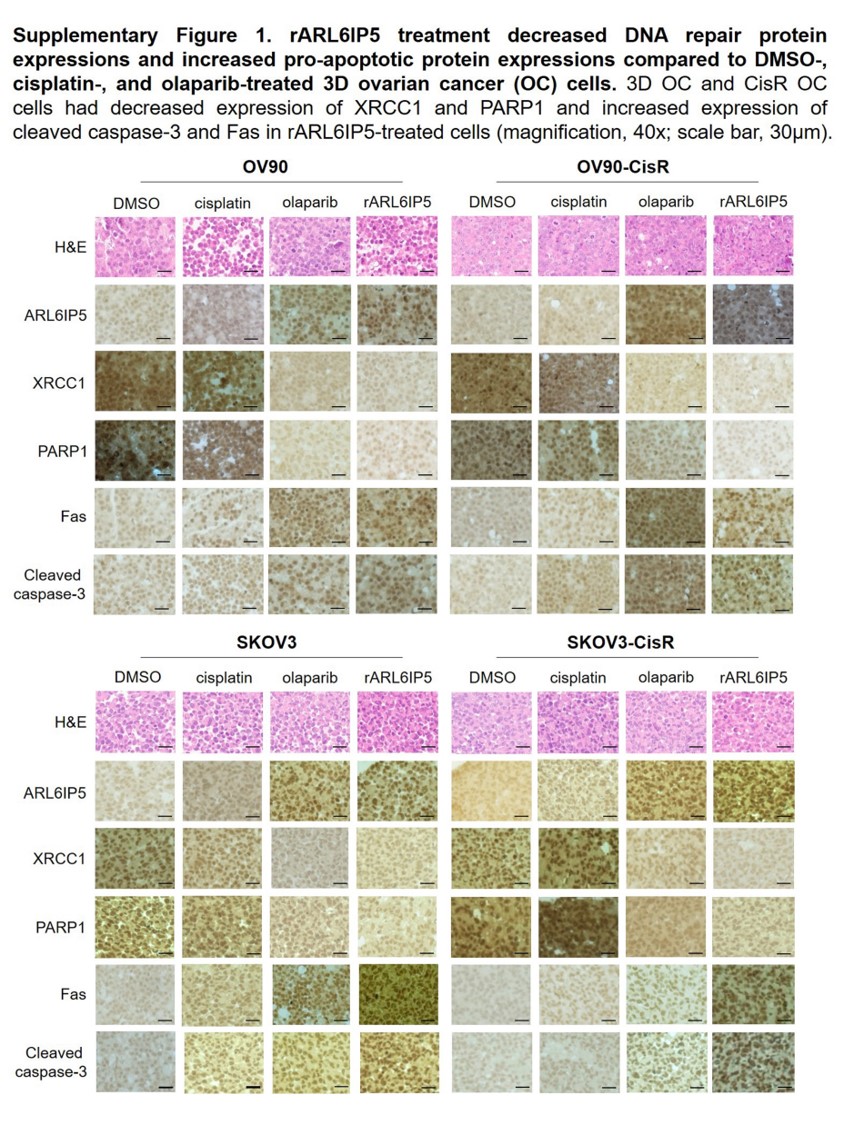

Supplement: Supplementary file 4 — Supplementary Figure 1 [file 41419_2022_4568_MOESM4_ESM.jpg]
