# Supplementary material for: ARL6IP5 reduces cisplatin-resistance by suppressing DNA repair and promoting apoptosis pathways in ovarian carcinoma
Source: Cell Death Dis. 2022 Mar 15;13(3):239. doi: 10.1038/s41419-022-04568-4 (PMC8924236; doi:10.1038/s41419-022-04568-4)
Supplement: Supplementary file 6 — Author Contribution Form [file 41419_2022_4568_MOESM6_ESM.pdf]

# DECLARATION OF CONTRIBUTIONS TO ARTICLE

**ADMC**

| | | |
|---|---|---|
| Manuscript Number: CDDIS-21-0205 | Journal Name: *Cell Death & Disease* | (the 'Journal') |
| Proposed Title of the Contribution: ARL6IP5 Reduces Cisplatin-Resistance by Suppressing DNA Repair and Promoting Apoptosis Pathways in Ovarian Carcinoma | | (the 'Contribution) |
| Author(s): Ji-Ye Kim, Entaz Bahar, Jung-Yun Lee, Sunhee Chang, Se Hoon Kim, Eun Young Park, Sung-Im Do, Hyonok Yoon, Hyun-Soo Kim | | (the 'Authors') |

For all *CDDis* articles, each person named as an author in the published version must be able to show he or she has contributed substantially to the article.

Authorship credit should be based on 1) substantial contributions to conception and design, acquisition of data, or analysis and interpretation of data; 2) drafting the article or revising it critically for important intellectual content; and 3) final approval of the version to be published. Authors should meet conditions 1, 2 and 3.

Any person who cannot be shown to have made a substantial contribution to the article cannot be listed as an author in the final version. The name of any person who is deemed to have made a minor contribution can, however, appear in the Acknowledgments section of the article.

Please complete the table below to indicate the contributions of all named authors to the manuscript.

| Author Full Name: | Specification of Contribution to the Manuscript: |
|---|---|
| Ji-Ye Kim | Conceptualization, data curation, formal analysis, funding acquisition, investigation, project administration, writing-original draft, review, and editing. |
| Entaz Bahar | Data curation, conceptualization, investigation, methodology, and formal analysis. |
| Jung-Yun Lee | Resources, data curation, and investigation. |
| Sunhee Chang | Pathologic interpretation. |
| Se Hoon Kim | Data curation. |
| Eun Young Park | Formal analysis. |
| Sung-Im Do | Methodology and editing. |
| Hyonok Yoon | Conceptualization, investigation, funding acquisition, project administration, and supervision. |
| Hyun-Soo Kim | Conceptualization, resources, funding acquisition, project administration, supervision, and writing-review and editing. |
| | |
| | |
| | |
| | |

Please complete the table below to indicate the contributions of all named authors to the figures.

Figure 1:

Ji-Ye Kim, Hyun-soo Kim, Jung-Yun Lee, Sunhee Chang, Se Hoon Kim

Figure 2:

Ji-Ye Kim, Hyun-soo Kim, Entaz Bahar, Hyonok Yoon

Figure 3:

Ji-Ye Kim, Hyun-soo Kim, Sung-Im Do, Entaz Bahar, Hyonok Yoon

Figure 4:

Ji-Ye Kim, Hyun-soo Kim, Entaz Bahar, Hyonok Yoon

Figure 5:

Ji-Ye Kim, Hyun-soo Kim, Entaz Bahar, Hyonok Yoon

Figure 6:

Ji-Ye Kim, Hyun-soo Kim, Entaz Bahar, Hyonok Yoon

| Signed for and on behalf of the Author(s): | Print Name: | Date: |
|---|---|---|
| Hyunsoo Kim | HYUN-SOO KIM | January 15th 2021 |
